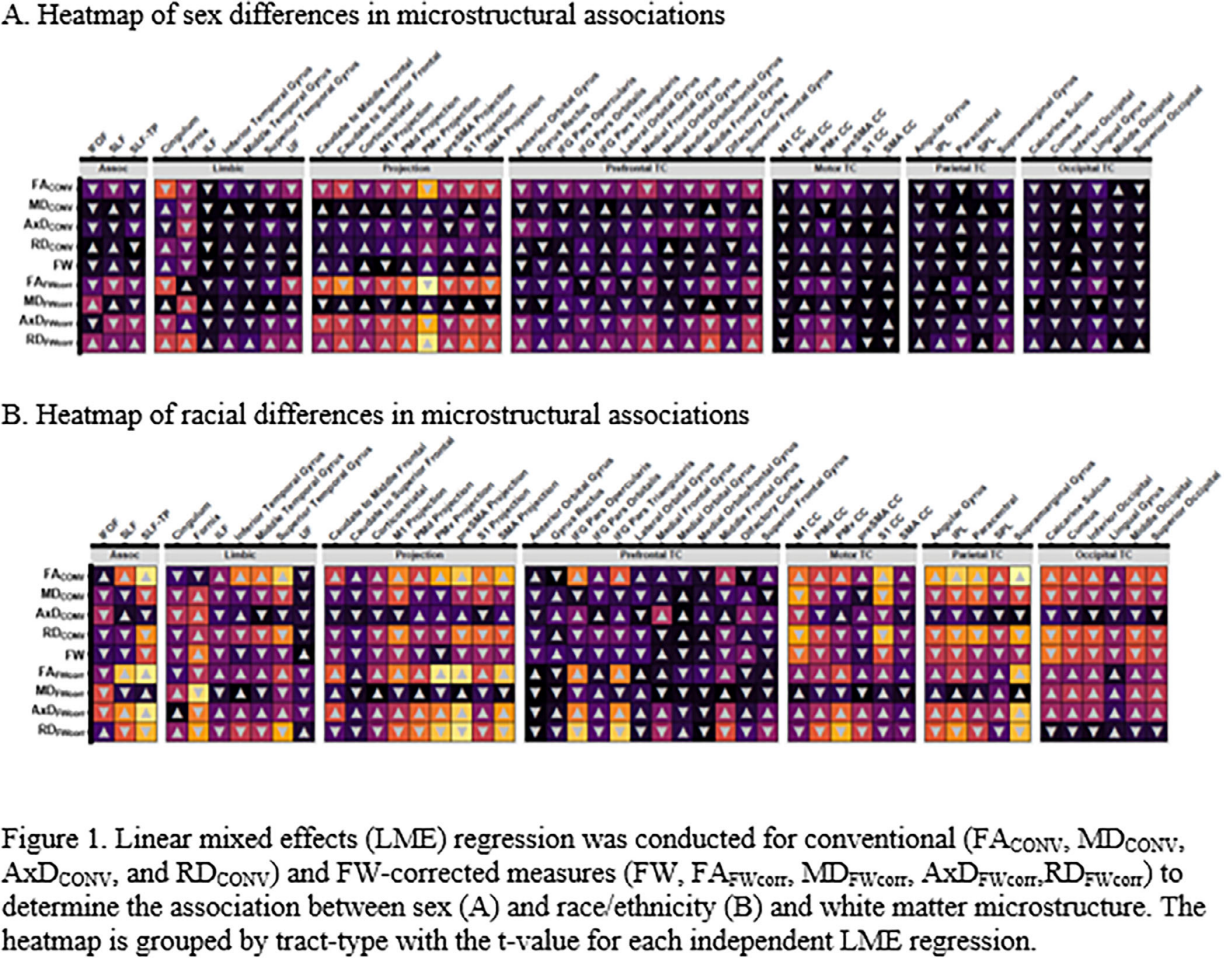# Sex, racial/ethnic, and APOE‐ε4 allele differences in longitudinal white matter microstructure in multiple cohorts of aging

**DOI:** 10.1002/alz.090505

**Published:** 2025-01-09

**Authors:** Amalia Jo Peterson, Aditi Sathe, Yisu Yang, Alaina Durant, Niranjana Shashikumar, Kimberly R. Pechman, Logan C. Dumitrescu, Katherine A. Gifford, Shannon L. Risacher, Lori L Beason‐Held, Yang An, Kurt Schilling, Bennett A. Landman, Julie A. Schneider, Lisa L. Barnes, David A. Bennett, Angela L. Jefferson, Susan M Resnick, Andrew J. Saykin, Timothy J. Hohman, Derek B. Archer, the Alzheimer's Disease Neuroimaging Initiative (ADNI)

**Affiliations:** ^1^ Department of Neurology, Vanderbilt University Medical Center, Nashville, TN USA; ^2^ Vanderbilt Memory & Alzheimer’s Center, Vanderbilt University Medical Center, Nashville, TN USA; ^3^ Vanderbilt Memory & Alzheimer's Center, Nashville, TN USA; ^4^ Vanderbilt Memory and Alzheimer’s Center, Vanderbilt University Medical Center, Nashville, TN USA; ^5^ Vanderbilt Genetics Institute, Vanderbilt University Medical Center, Nashville, TN USA; ^6^ Indiana University School of Medicine, Indianapolis, IN USA; ^7^ Center for Neuroimaging, Department of Radiology and Imaging Sciences, Indiana University School of Medicine, Indianapolis, IN USA; ^8^ National Institute on Aging, Baltimore, MD USA; ^9^ Vanderbilt University Institute of Imaging Science, Vanderbilt University Medical Center, Nashville, TN USA; ^10^ Department of Radiology & Radiological Sciences, Vanderbilt University Medical Center, Nashville, TN USA; ^11^ Department of Biomedical Engineering, Vanderbilt University, Nashville, TN USA; ^12^ Department of Electrical and Computer Engineering, Vanderbilt University, Nashville, TN USA; ^13^ Vanderbilt University Institute on Imaging Science, Vanderbilt University Medical Center, Nashville, TN USA; ^14^ Rush Alzheimer’s Disease Center, Rush University Medical Center, Chicago, IL USA; ^15^ Rush Alzheimer's Disease Center, Rush University Medical Center, Chicago, IL USA; ^16^ National Institute on Aging, NIH, Baltimore, MD USA; ^17^ Indiana Alzheimer's Disease Research Center, Indianapolis, IN USA; ^18^ Indiana University, Indianapolis, IN USA

## Abstract

**Background:**

There is growing recognition that white matter microstructural integrity is affected in Alzheimer’s disease. The goal of this study was to characterize sex, racial/ethnic, and apolipoprotein (APOE)‐ε4 allele differences in white matter integrity.

**Methods:**

This study included participants from ADNI, BLSA, ROS/MAP/MARS, and VMAP, all longitudinal cohorts of aging. This combined dataset included 6,837 imaging sessions from 2,619 participants age 50+ with diffusion MRI (dMRI) and demographic and clinical data (60% female, 31.4% APOE‐ε4 carriers, 78.9% White). dMRI was preprocessed using the PreQual pipeline. Free‐water (FW) correction was used to generate FW and FW‐corrected intracellular metrics including fractional anisotropy (FA_FWcorr_), mean diffusivity (MD_FWcorr_), axial diffusivity (AxD_FWcorr_), and radial diffusivity (RD_FWcorr_). Conventional and FW‐corrected metrics were harmonized using the Longitudinal ComBat package. Linear mixed‐effects models related sex, race/ethnicity, and APOE‐ε4 allele status to longitudinal diffusion metrics in 48 white matter tracts, adjusting for age at baseline, sex, education, race/ethnicity, APOE‐ε4 carrier status, cognitive status at baseline, and converter status. All models were corrected for multiple comparisons using the FDR approach.

**Result:**

Sex differences in white matter were most notable in projection tracts (Figure 1A) and were primarily in FW‐corrected metrics. Females had lower FA_FWcorr_ and higher RD_FWcorr_, indicative of worse microstructure, but lower AxD_FWcorr_. This sex difference was most pronounced for FA_FWcorr_ in the ventral premotor projection tract (p=1.53x10^‐62^). There were global differences in white matter integrity by race/ethnicity (Figure 1B). Non‐Hispanic White participants tended to have higher conventional FA, FA_FWcorr_ and AxD_FWcorr_ and lower RD_FWcorr_. There was no association between APOE‐ε4 status and white matter integrity and no significant sex x race/ethnicity, sex x APOE‐ε4, or race/ethnicity x APOE‐ε4 interactions for conventional or FW‐corrected metrics when corrected for multiple comparisons.

**Conclusion:**

There were striking sex and racial/ethnic (but not APOE‐ε4) differences in white matter tract integrity in a large cohort of aging adults. Female participants tended to have measures reflective of worse white matter integrity, and non‐Hispanic White participants tended to have measures reflective of greater integrity. Additional research exploring the etiology of these differences will be important to better understand disparities in Alzheimer’s disease.